# What Approaches to Thwart Bacterial Efflux Pumps-Mediated Resistance?

**DOI:** 10.3390/antibiotics11101287

**Published:** 2022-09-21

**Authors:** Armel Jackson Seukep, Helene Gueaba Mbuntcha, Victor Kuete, Yindi Chu, Enguo Fan, Ming-Quan Guo

**Affiliations:** 1 CAS Key Laboratory of Plant Germplasm Enhancement and Specialty Agriculture, Wuhan Botanical Garden, Chinese Academy of Sciences, Wuhan 437004, China; 2 Department of Biomedical Sciences, Faculty of Health Sciences, University of Buea, Buea P.O. Box 63, Cameroon; 3 Sino-Africa Joint Research Center, Chinese Academy of Sciences, Wuhan 437004, China; 4 Innovation Academy for Drug Discovery and Development, Chinese Academy of Sciences, Shanghai 201203, China; 5 Department of Biochemistry, Faculty of Science, University of Dschang, Dschang P.O. Box 67, Cameroon; 6 State Key Laboratory of Medical Molecular Biology, Department of Microbiology and Parasitology, Institute of Basic Medical Sciences, Chinese Academy of Medical Sciences/School of Basic Medicine, Peking Union Medical College, Beijing 100005, China; 7 College of Life Sciences, Linyi University, Linyi 276005, China

**Keywords:** antibiotics, antimicrobial resistance, efflux pumps, multidrug resistance, antibiotic resistance breakers, efflux pump inhibitors, membrane permeabilizers

## Abstract

An effective response that combines prevention and treatment is still the most anticipated solution to the increasing incidence of antimicrobial resistance (AMR). As the phenomenon continues to evolve, AMR is driving an escalation of hard-to-treat infections and mortality rates. Over the years, bacteria have devised a variety of survival tactics to outwit the antibiotic’s effects, yet given their great adaptability, unexpected mechanisms are still to be discovered. Over-expression of efflux pumps (EPs) constitutes the leading strategy of bacterial resistance, and it is also a primary driver in the establishment of multidrug resistance (MDR). Extensive efforts are being made to develop antibiotic resistance breakers (ARBs) with the ultimate goal of re-sensitizing bacteria to medications to which they have become unresponsive. EP inhibitors (EPIs) appear to be the principal group of ARBs used to impair the efflux system machinery. Due to the high toxicity of synthetic EPIs, there is a growing interest in natural, safe, and innocuous ones, whereby plant extracts emerge to be excellent candidates. Besides EPIs, further alternatives are being explored including the development of nanoparticle carriers, biologics, and phage therapy, among others. What roles do EPs play in the occurrence of MDR? What weapons do we have to thwart EP-mediated resistance? What are the obstacles to their development? These are some of the core questions addressed in the present review.

## 1. Introduction

How can antimicrobial resistance (AMR) be prevented or reversed? This subject is frequently raised in scientific alerts. The scientific community is discussing novel and groundbreaking ideas to address the renewed outbreak of antibiotic-resistant infections worldwide. AMR is a worldwide disaster; the figures are worrisome, and estimates do not portend positive outcomes. Indeed, according to O’Neil [1], in the absence of effective and timely interventions to stop the alarming expansion of AMR, the disturbing 700,000 deaths annually due to hard-to-treat infections will rise to 10 million per year by 2050. Today, such a sharp escalation is already apparent, as evidenced by a recent and thorough assessment of the AMR load [2]. Using a predictive statistical model covering 204 countries and territories with 471 million unique records or isolates, Murray and co-workers [2] quoted the death toll of 4.95 million (in 2019) due to bacterial AMR, the highest death rate (at 27.3 deaths per 100,000) being in western sub-Saharan Africa. Six pathogenic bacteria have been identified so far as the leading cause of AMR-related deaths, namely *Streptococcus pneumoniae, Staphylococcus aureus, Pseudomonas aeruginosa, Escherichia coli*, *Acinetobacter baumannii*, and *Klebsiella pneumoniae* [2]. As such, the data are pointing to an impressive trend towards increased resistance, moving towards a risk of therapeutic deadlock. A 2021 World Health Organization (WHO) report [3] portrays the antibacterial pre- and clinical development pipeline as stagnant and lagging far behind current global needs. As of 2017, merely 12 antibiotics have been approved, out of which 10 were from already known classes with evidence of AMR mechanisms. According to the same WHO report, in 2021, as compared to 31 products in 2017, only 27 novel antibiotics were in clinical development against the most dangerous form of antibiotic-resistant bacteria (priority pathogens described above). Looking more generally, the WHO analysis shows that among the 77 agents against bacteria in clinical development, there were 45 direct-acting conventional small molecules and 32 unconventional agents [3]; this analysis further confirms the increasing paucity of innovative (new chemical structure, uncommon mode of action, non-toxic) and effective antibacterial agents. 

Although countless efforts are being made by both national and international organizations as well as scientists to tackle AMR, it remains a serious threat to public health, because the root cause is hard to control. The underlying cause is the inappropriate and abusive use of antibiotics in both human and veterinary medicine [4,5,6]. Moreover, the inherent ability of most pathogenic bacteria to resist any substance that might interfere with their survival, including antibiotics, is a major factor contributing to this phenomenon. Over time, bacteria have elaborated several ways to surmount the inhibitory or killing effects of classical antibiotics [7]. The over-expression of efflux pumps (EPs) has emerged as the primary mechanism of multidrug resistance (MDR) development in bacteria [8]. The EPs are transmembrane proteins used by bacteria to extrude any substance harmful to their survival; these pumps can reject a wide range of compounds with different chemical structures, including antibiotics. Therefore, in bacteria, active efflux appears to be a defense mechanism. There are five major families of EPs that have been established to date: ATP Binding Cassette (ABC), Multidrug and Toxin Extrusion (MATE), Small Multidrug Resistance (SMR), Major Facilitator Superfamily (MFS), and Resistance Nodulation Cell Division (RND) [9]. Besides these common transporters, one additional efflux protein has been characterized from *A. baumannii* designated PACE, which stands for Proteobacterial Antimicrobial Compound Efflux. The latter is structurally related to the SMR family of transporters [10]. The distinction between the various family is determined by the source of energy used to operate the pump. For instance, ABC uses the energy derived from ATP degradation (therefore known as primary active transporters), while the others (secondary active transporters) rely on the energy from the electrochemical gradient by either H^+^ (proton motive force, e.g., RND, SMR, and MFS) or Na^+^ (sodium motive force, e.g., MATE) [9]. The complex nature of the organization of EPs also revealed the organizational and molecular pathways of transport of the substrate. In Gram-positive bacteria (GPB), the drug resistance is driven primarily by EPs localized in the cytoplasmic membrane, whereas Gram-negative bacteria (GNB) EPs have greater complexity as a result of their double-layered cell membranes, namely the internal or cytoplasmic membrane and the outer membrane, and they are segregated by the periplasmic space, creating a three-layered protein channel that mediates extrusion of the drug. To illustrate, the RND family EPs are organized in a tripartite shape and are predominant promoters of inherent antibiotic resistance in GNB, exporting a broader spectrum of antibiotics and biopharmaceuticals [11]. Notwithstanding, in GPB, predominantly MFS transporters include NorA, EmeA, and PmrA, respectively from *S. aureus*, *E. faecalis*, and *S. pneumoniae* that expel a wide range of various classes of antibiotics [12]. Contrary to many other resistance promoters, EPs are more often intrinsic. Both sensitive and resistant bacteria have genes encoding the efflux transporters [13], which are frequently a component of a transcriptionally controlled operon. Further to regulatory protein or promoter mutations, these EPs are overexpressed, leading to drug resistance [13]. Mechanisms of efflux in bacteria can be either specific or nonspecific. When specific, the pump recognizes and pumps out a unique antibiotic molecule or a single antibiotic class, for instance, TetA pumps exclusively extrude tetracycline. Another example is AbaF, specific to fosfomycin. Otherwise, the efflux machinery is capable of extruding more than one class of antibiotics, for instance, MexAB-OprM, NorA, and BmrA that expel a wide range of substrates [14]. The latter is referred to as MDR EPs. In addition to the resistance to drugs, the bacterial physiological role of EPs expands to cover bile tolerance in Enterobacteriaceae, resulting in colonization, enhanced virulence, biofilm formation, and host bacterial survival [15]. Moreover, EPs are also implicated in bacterial communication processes including quorum sensing [8,16], in which interconnection contributes to the further propagation of resistant phenotypes.

Considering the current critical role of EPs in the emergence of MDR, its inhibition has been widely recognized and validated as an efficient and long-term control of bacterial resistance. Any compound that specifically targets and blocks EPs is referred to as an efflux pump inhibitor (EPI). There is evidence that the inhibition of EPs is an intriguing approach as the supply of new antibiotics dries up. EPIs adhere to certain common inhibitory mechanisms of efflux and are being developed from a variety of synthetic and naturally-occurring sources [8,17,18]. Prospects for the use of EPIs include the possibility of preventing the occurrence of resistance, as well as the reuse of old antibiotics, which were previously discarded after losing efficiency owing to bacterial resistance. Hence, as emphasized by Spengler et al. [19], we are indeed moving in new directions leading to old destinations. What roles do EPs play in the occurrence of MDR? What weapons do we have to thwart resistance via active efflux? What are the obstacles to their development? These are some of the core questions addressed in the present review.

## 2. Efflux Pump-Mediated Multi-Drug Resistance in Bacteria

### 2.1. Underlying Biochemical Basis of Bacterial Resistance: An Overview

Resistance to antimicrobials (AMR) is an inherent event and an integral part of the intrinsic properties of bacteria. In theory, resistance occurs when bacteria are exposed to antibiotics, causing selective pressure and disrupting the balance between resistant and susceptible bacterial populations. Although natural, this phenomenon has been expanding rapidly over the years. The root determinants are the unrestricted and improper utilization of antimicrobial drugs in both human and veterinary medicine; this has led to acquired resistance, resulting in the advent of multidrug-resistant (MDR) bacteria. Transferring genes horizontally and/or spontaneous chromosomal mutations are the main factors generating acquired resistance [20,21]. The genetic elements, which include plasmids, transposons, and integrons, actively participate in the transfer of genes for resistance horizontally from one bacterium to another; this phenomenon can happen within the same or different bacterial species, and it represents one of the prevailing drivers of MDR [21].

Several defense strategies have been developed by bacteria to escape antibiotic effects. (i) Invisibility cloak The bacteria modify their physiology to prevent the antibiotic from finding its intended target. The cell wall structure is actively modified by some bacteria to render it invisible from the outside. (ii) Counterattack In some cases, bacteria release protective enzymes that either degrade or modify the drug’s molecule and cause them to be completely ineffective. (iii) Protein shield Bacterial proteins bind to antibiotics or the drug target in the bacterial cell. Therefore, this protein hinders the effective bonding of the antibiotic to its specific target. (iv) Pump and flush Bacteria commonly expel noxious agents out of their system through a transmembrane protein (EP) that is required to export them outside the cell. The efflux machinery has evolved to actively remove antibiotics. Other underlying biochemical mechanisms of AMR in bacteria include porin loss (which lead to the reduced passage of antibiotic in the cell) and biofilm formation [21,22]. The present review emphasizes the role of EPs in the occurrence of MDR.

The EPs operate as a primary protective barrier against antimicrobial drugs by diminishing the intracellular distribution of the drugs; this defensive barrier involves a variety of transport proteins, lodged both within the cell membrane and the periplasmic space, which expels a range of exogenous and noxious compounds out of the bacterial cells [23]; this means that bacterial EPs do not simply perform the role of physiological transport, their function is also to compete for survival under external influences. Though certain EPs have unique substrates, some carriers can handle many different types of antimicrobials, thereby promoting MDR [24]. Occasionally, extruding organic solvents or other substrates results in the over-expression of transporters, generating a co-selection of AMR traits [25]. Over-expression of EPs is also predicted to influence the pathogenic characteristics of bacteria, entailing the quorum signaling and the formation of biofilms [26]. Thus, EPs do not only export antimicrobials but also determinants of virulence [15]. 

**Figure 1:**
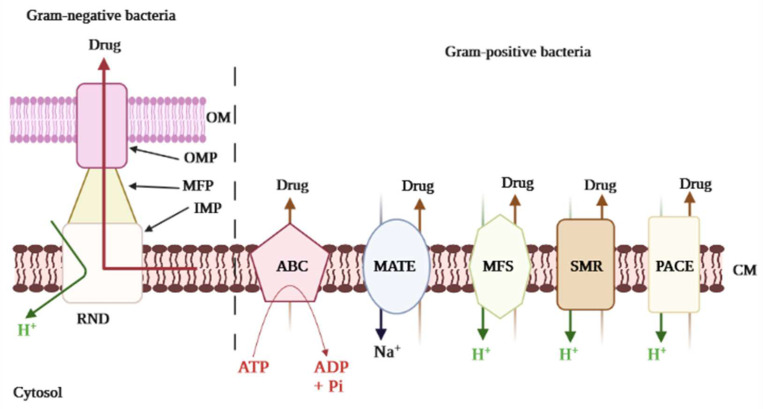
Major efflux pump systems in bacteria. The major efflux pump family, namely ABC (ATP Binding Cassette), MATE (Multidrug and Toxic Compound Extrusion), MFS (Major Facilitator Superfamily), SMR (Small Multidrug Resistance), RND (Resistance Nodulation and Cell Division), and PACE (Proteobacterial Antimicrobial Compound Efflux) are represented. The specificity of Gram-negative bacteria is shown with the presence of the OM (Outer Membrane) in addition to the CM (Cytoplasmic Membrane). The components of RND are OMP (Outer Membrane Protein), MFP (Membrane Fusion Protein), and IMP (Inner Membrane Protein), making the tripartite conformation. The primary transporters use energy derived from ATP hydrolysis (ABC), whereas the secondary transporters use the energy from the H^+^ motive force or the Na^+^ electrochemical gradient.

Extensive literature has detailed EP-mediated resistance pathways, and increasingly, more novel EPs and associated proteins are being uncovered. Still, the specific mechanics and functional domains of EP transporters remain unclear. Numerous conditions exist that impact the bacterial inner and outer membrane (OM), promote pump activity and favor structural changes of the transporters in the fluid membrane environment [23]. Regardless, a recent study by Krishnamoorthy et al. [27] displayed an interaction between active EPs and the OM permeability barrier in *Burkholderia thailandensis*, a non-fermenting motile, Gram-negative bacillus. The major groups of the EPs superfamily (Figure 1) are ABC, MFS, MATE, SMR, and RND. A sixth category was unveiled a few years ago, namely PACE [28]; this suggests a progressive quest into the detection and characterization of MDR EPs in bacteria.

**Figure 2:**
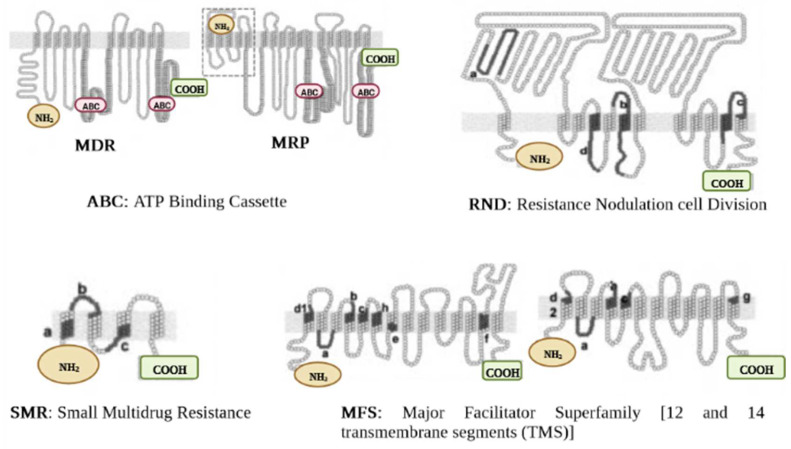
Characteristic topology of major efflux pumps. Proteins are shown as a chain of circles, with solid lines (indicated by letters from a to g) representing conserved motifs in RND, SMR, and MFS superfamilies. MDR, multi-drug resistance. MRP, multi-drug resistance protein.

**Figure 3:**
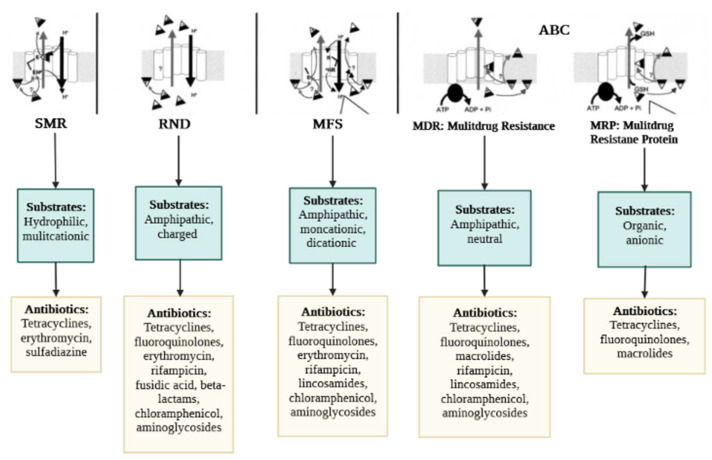
Mechanistic pathways and major substrates (antibiotics) of bacterial efflux pumps. Mechanistic pathways: The proton (H^+^) antiporter mechanism is used by the SMR, MFS, and RND pumps. H^+^ is most likely transported from glutamate (E) in the “a” conserved segment of SMR and from arginine (R) in the “b” conserved portion of MFS (these domains are identified in Figure 2). The same conserved amino acid (E) for SMR pumps and a conserved amino acid residue (E or aspartate) in the “d” region for MFS proteins may suggest recognition of positively charged substrates. ATP is used as an energy source by ABC pumps. With all families of pumps, the target drug appears to be removed from the membrane instead of the cytosol. The carrier might then act as a flippase, speeding the flow of the chemical from the membrane’s inner to the outer surface. Alternatively, the pump might act as an "aspirator," vacuuming the chemical from the membrane and transporting it to the inner region of a channel that is closed to the cytosolic section of the membrane but accessible to its outer surface. MRP transporters (ABC) also need glutathione (GSH), which can be coupled to the drug before or during extrusion. In addition, the MRP transporters (ABC) rely on glutathione (GSH), which can be coupled to the substrate either before or during the extraction process. Common substrates and antibiotics: Chemical classes of antibiotics for which efflux has been documented for one or more pumps in each family are grouped according to their general physicochemical properties.

The classification of the EPs is determined according to the type of energy they use to operate. Thus, the primary transporters use energy derived from ATP hydrolysis (e.g., ABC), whereas the secondary transporters benefit from the energy derived from the H^+^ motive force or the Na^+^ electrochemical gradient (e.g., MFS, MATE, SMR, RND) [29]. Besides, some heterogeneities were revealed in the components of the efflux proteins. For example, RND, unlike other pumps, has three major components consisting of an outer membrane channel protein (OMP), the inner membrane transporter (IMP), and a membrane fusion protein (MFP); this gives the protein a tripartite shape (Figure 1). The OMP and IMP are joined together by MFP, and the assembly pumps out antibiotics and other noxious substances [30]. Over-expression of EPs is an underlying factor in the emergence of MDR. The molecular organization of EPs and their critical drug-binding sites are fundamental to the successful delivery of EP inhibitors (EPIs). We have reviewed the structures of the different EP families for both GNB and GPB and briefly discuss them further below. The characteristic topology of the major EPs is presented in Figure 2, whereas the mechanistic pathways and major substrates (antibiotics) are depicted in Figure 3.

### 2.2. Efflux Pumps Superfamilies and Their Role in Imparting Multidrug Resistance in Bacteria

#### 2.2.1. ABC Superfamily

The structure of ABC pumps differs between GPB and GNB. ABC protein in GPB is made up of one transmembrane protein. Typical examples include EfrAB, LmrA, Msr, and PatA/B. EfrAB expressed in *Enterococcus faecalis* [31], is a heterodimeric MDR pump, with common substrates including aminoglycosides (e.g., streptomycin, gentamicin) and phenicole (e.g., chloramphenicol). EfrAB expression is driven significantly by the sub-MIC values of such antibiotics [32]. The LmrA protein has been characterized in *Lactococcus lactis* [31]. The pump operates similarly to a homodimer, consisting of a trans-membrane (made up of six alpha helix) and single nucleotide-binding domains. Macrolides and lincosamides are the main compounds recognized and transported by the pump [33]. The transmembrane domain is missing in Msr detected from *Streptococcus*, however, unlike LmrA, two nucleotide-binding domains are found in the organization of Msr; this characteristic lends itself to macrolide resistance [34,35]. The PatA/B is another MDR pump from *Streptococcus*. It has been reported to target (efflux) all of the hydrophilic fluoroquinolone class of antibiotics including ciprofloxacin and norfloxacin [36]. 

The most thoroughly explored ABC-related transporter in GNB is the MacAB-TolC. The EP has a tripartite complex structural configuration (which consists of an IMP MacB, MFP MacA, and OMP TolC) which actively extricates substrates such as macrolides and virulence factors (polypeptides) through MacB ATPase [37,38]. Other physiological substrates of MacAB-TolC comprise the lipopolysaccharide (LPS) or related glycolipids [38]. The IMP MacB also operates as a homodimer complex. A nucleotide-binding motif at the N-terminus binds ATP, and a cytoplasmic tail at the C-terminus, whereas MacA (MFP) connects directly to the LPS core and is activated by ATPase [38]. Therefore, this tripartite complex organization composed of the TolC (OMP), MacB (IMP), and MacA (periplasmic protein) constitutes the topological location for substrate transport [37]. Shirshikova et al. [39] demonstrated how the absence of a MacAB pump in *Serratia marcescens* promotes vulnerability towards polymyxins and aminoglycosides, decreases motile swimming, and potential to form biofilms, and finally culminates in decreased superoxide stress capacity. Furthermore, MacAB also mediates antibiotic resistance in *Agrobacterium tumefaciens* to penicillin and As (III) [40].

#### 2.2.2. MFS Superfamily

This family is the most extensive transporter group ever characterized, predominantly in GPB. Structurally, the EPs membership of this superfamily is formed by 12 or 14 transmembrane regions (Figure 2) and are largely involved in MDR [41]. Some examples include Lde efflux proteins (detected from *Listeria monocytogenes*) [42] and another common pump, NorA (detected from *S. aureus*) [43], which exclusively expel fluoroquinolones (ciprofloxacin, norfloxacin), whereas macrolides are extruded by Mef (detected in *S. pneumoniae*) [44]. Chancey et al. [45] further pointed out that the initiation of *mef(E)/mel* gene transcription takes place in the *mef(E)/mel* promoter, whereby the decrease in transcription was found to influence the regulation of macrolide resistance mediated by mef. In further development, Msr protein (ABC family) and Mef (MFS family) synergistically improve macrolide efflux, whereby resistance to 14- and 15-membered ring macrolides is enhanced [46].

In tandem with drug efflux, the MFS superfamily also serves valuable regulatory functions in a variety of biological processes. For instance, some pumps including MdrM and MdrT participate in fostering the response of the host immune system through activation of Interferon beta (IFN-β) generation (interferon type I response) and maintain the integrity of the cell wall [47]. Similarly, the Tet38 pump affects several steps in the pathogenic process of *S. aureus* invasion into host cells, involving adherence, penetration, and transport into the epithelium. The subsequent stage involves bacterial viability and transport into phagolysosomes [47]. AbaQ, another member of the MFS superfamily is implicated in *A. baumannii* pathogenicity. It has been demonstrated that the absence of abaQ gene lowers the motility of *A. baumannii* as well as virulence [47]. Furthermore, disabling the genes that code for the EPs including MATE, RND, SMR, and ABC also decreases those two pathogenic factors when compared to the parental strain [48]; this exemplifies the interconnection that exists between efflux proteins and pathogenic factors, resulting in increased resistance to therapeutics.

#### 2.2.3. MATE Superfamily

Structurally, members of this superfamily are composed of 12 transmembrane domains (alpha helix) [41]. The sequence similarity of amino acids (AA) is used to classify MATE transporters. Typical classes include DinF, NorM, and others found in eukaryotic cells [49]. MATE family transporter substrates are varied and exhibit different chemical structures, yet fluoroquinolones are found to be the substrates that nearly all transporters recognize. The most studied MATE pump in GNB is NorM [50]. Investigations on EPs from *N. gonorrhoeae* depicted the significant efflux ability of NorM towards ciprofloxacin, solithromycin, and positively charged antimicrobial compounds including quaternary ammonium [51]. Moreover, a distinct capacity to extradite intracellular ROS (reactive oxygen species) has been ascribed to NorM, mitigating potential harm from oxidative stress [52]. Otherwise, in *Pneumococci*, greater susceptibility to quinolone/fluoroquinolone antibiotics was observed as a result of DinF transport machinery mutations [53]. The following genes, cinA, recA, and dinF, encode for a competence-induced protein A, which together form a quinolone-induced operon via the SOS response [53].

#### 2.2.4. SMR Superfamily

Structurally, members of this superfamily consist of small polypeptides, with the sequence of AAs ranging between 100–150 molecules; they are shaped as four transmembrane α-helices spanning the cytoplasmic membrane [54]; these short hydrophilic loop proteins facilitate the solubilization of a wide range of structurally diverse drugs [54]. For instance, AbeS pump expressed in GNB *A. baumannii* is involved in the efflux of amikacin as well as some antiseptic compounds including acriflavine and benzalkonium [55]. Similarly, KpnEF pump detected from *K. pneumoniae* clinical strains also depicted higher resistance to some drugs of the class of antiseptics [56]. The protein EmrE, found in *P. aeruginosa* and *E. coli*, recognizes and drives toxic polyaromatic agents’ extrusion [57]. Another protein, Qac, is also reported to influence the resistance to certain antibiotics and antiseptics [58]. Gene qacA/B is often characterized in some GPB (*S. aureus, E. faecalis*), while gene qacE is broadly shared in *Pseudomonas spp.* and Enterobacteriaceae [58]. SMR protein-coding genes are often located on integrons and MDR plasmids, which enhances the likelihood of horizontal gene transmission [59]. The over-expression of EPs initiated by QAC exposure mediates the horizontal transfer of integrons harboring qnr, aac(60)-Ib-cr, oqxAB, qepAB (FQ resistance determinants) into QacED1, a class 1 integrons [60]. When disinfectant resistance and AMR genes are concomitantly transferred between distinct species, it severely influenced the killing effects of both class compounds.

#### 2.2.5. RND Superfamily

Typically, RND EPs are expressed in GNB, expelling a large range of antibiotics and noxious chemicals. Some well-characterized RND pumps involve AcrAB-TolC, AdeABC, MexAB-OprM, respectively from *E. coli*, *A. baumannii*, and *P. aeruginosa* [61,62,63]. Other pumps including CmeABC, MtrCDE, OqxAB, SmeABC, TtgABC have been reported, respectively in *Campylobacter jejuni*, *N. gonorrhoea*, *K. pneumoniae,* and *Salmonella enterica*, *Stenotrophomonas maltophilia*, and *Pseudomonas putida* [62,64,65,66,67,68]. Internal transport proteins (e.g., AdeB, CmeB, MexB, SmeB, MtrD, and TtgB) are involved in the substrate-specific binding and carriage of diverse drug categories, serving essential functions in clinically relevant resistance [69]. As an example, AcrB gene mutations are believed to cause ciprofloxacin treatment failure [70]. The above EPs expression is also being regulated at the transcriptional level by regulatory proteins that belong to the TetR family, including AcrR [71], CmeR [72], NalC/NalD [73], TtgR [74] SmeT [75], and MtrR [76], as well as MexR of the MarR family [77]. The AA residues in EPs can serve as critical sites for the binding of substrates, and the switching of AA residues is likely to alter the substrate affinity [78]. Besides, changes in salient residues of AA proved to correlate with EP-mediated drug resistance [79]. Some well-characterized MDR EPs membership of the RND family comprising MexAB-OprM, MtrCDE, AcrAB-TolC, CmeABC are important for bacterial survival and pathogenicity. It has been shown in several investigations that the AcrAB-tolC EP affects the attachment of bacteria and penetration into the cells of the host as well as proliferation and persistence in animals [15]. Heavy metals (HME) have been widely applied in antimicrobials such as disinfectants. The RND superfamily in *E. coli* has an active contribution to antibiotic and HME resistance. The RND proteins have been classified into two major categories. The first group constitutes the HAE-RND family (hydrophobic and amphiphilic RND system) including AcrD, MdtB, AcrF, AcrB, MdtC, and YhiV, while the second group is a component of the HME-RND system. A typical model of such a system is CusA, with Ag(I) and Cu(I) being the major substrates [80]. CusA, MFP CusB, and channel protein CusC combine to produce the CusCBA tripartite efflux complexes [81]. The mechanistic pathways of metal ions export have been predicted [81]. EP-mediated biodegradation is how bacteria prevent themselves from the harmful effects of toxic components derived from organic pollutants. The TtgABC pump is responsible for toluene tolerance in *P. putida* [82]. In the RND family, the stress induced by antimicrobial abuse augments the event of functional mutations, which may reinforce its efflux potential. The EP substrate specificity is correlated with differences in AA residues in the binding site, and bacteria become less responsive to antimicrobials after the replacement of these AAs. Many cases of genetic changes in the RND pumps that have been previously determined in isolates/strains from clinical, environmental, and laboratory collection significantly affect AMR [83]. A genetic change in an EP could represent an evolutionary adaptation of microorganisms to antimicrobial drugs, so it undoubtedly complicates the overall therapeutic control of pathogenic bacteria.

#### 2.2.6. PACE Superfamily

PACE transporters have been identified recently, with Acel reported as the earliest member [84]. Acel has been characterized from *A. baumannii*, and the transporter helps in the biosynthetic biocide extrusion including quaternary ammonium antimicrobial agents (e.g., dequalinium, benzalkonium) and some other antiseptics including chlorhexidine, proflavine, acriflavine [28]. The PACE family of coding genomes has been found to remain highly conserved throughout bacterial species, implying that genes associated with PACE transporters are perpendicularly acquired and keep their functionality across host species [85]. AceI protein is analogous to SMR family members in both its predetermined secondary structure and size. It incorporates two tandem bacterial transmembrane pairs. AceI and its homologous domain proteins (e.g., BTP) were also unveiled in several pathogens comprising *Enterobacter, Salmonella*, *Pseudomonas*, *Burkholderia*, and *Klebsiella* [85]. An equilibrium pattern exists between a monomer and a dimer in the structure of the AceI pump. Increased chlorhexidine concentration and higher pH favor the acetylated dimer to form, while the coupling of chlorhexidine to AceR transcriptional protein increases the transcription of AceI, causing it to extrude chlorhexidine [84].

### 2.3. Expression of Efflux Pump Gene, Detection of Antimicrobial Resistance, and Clinical Therapy

Bacterial MDR EP genes elicit either innate or acquired AMR. The genes associated with resistance codetermine the constitutive or the EP regulatory proteins, providing the first line of defense against drugs and ensuring the persistence of bacteria [86]. Many EP genes can be employed for quick identification of AMR, which can effectively be ascertained by polymerase chain reaction (PCR) identification and establishing the MIC. A wide range of substrates is recognized by the RND EP family, resulting in most drugs being extruded and increasing AMR. Some RND representatives comprise Ade, Acr, and Mex pumps expressed in *A. baumannii*, *E. coli*, and *P. aeruginosa*, respectively [55]. The expression of the gene responsible for efflux can be critical in determining AMR and in guiding clinic-based therapy [87]. To illustrate, in an isolate of *A. baumannii* resistant to carbapenems, there was a multi-fold increase in efflux gene expression [88], involving *adeG*, *adeB*, and *adeJ*. In addition, enhanced expression of *adeB* and *adeJ* also occurred in bacteria withstanding tetracycline [89]. Yet, there exists some degree of correlation between substrate concentration and rate of peak transport. Thus, for instance, though cefaloridin may show high efflux through AcrB, the compound retains its antibacterial potential when AcrB is present, so the effective dose of cefaloridin is considerably weaker than the requisite efflux concentration [90]. Besides, certain EPs, which selectively bind a unique substrate were found to be causally related to significant resistance and MIC levels. Some examples of these pumps are MacAB, which selectively binds macrolides [44], OqxAB, which is specific to fluoroquinolones [91], and TetA/TetO which selectively expel tetracycline [92].

## 3. Strategies to Thwart Efflux Pump-Mediated Bacterial Resistance

**Figure 4:**
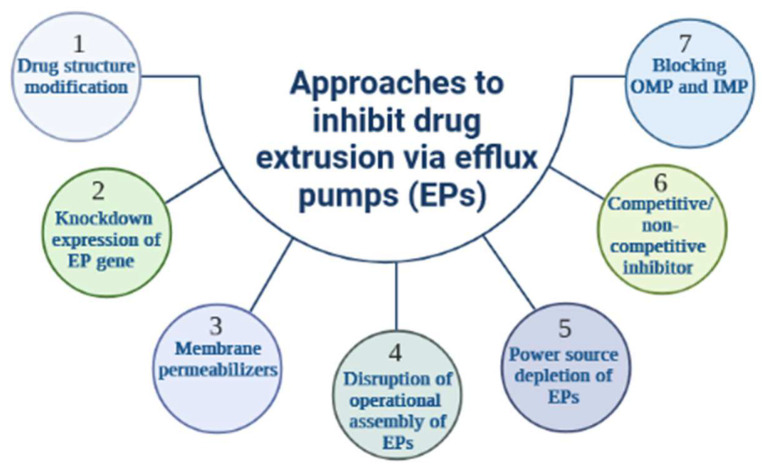
Approaches to counteract drug extrusion via efflux pumps. Several approaches to inhibit drug extrusion via efflux pumps (EPs) are described. Modification of drug structure results in a defect in drug recognition by EPs (1), whereas down-regulation of EP gene expression reduces EP activity and increases intracellular drug concentration (2), the latter also being achievable by the action of membrane permeabilizers (3). Some inhibitors can disrupt the functional assembly of EPs, thereby altering the efflux machinery (4). Since all EPs are sensitive to their energy source, a shutdown would significantly affect the energy level and subsequently reduces drug efflux (5). Competitive as well as non-competitive blockers have also been used (6) and they specifically block drug extrusion channels (7). In addition, some inhibitors can target the OMP (outer membrane protein) and IMP (inner membrane protein) of RND pumps.

The function of EPs may be evaded or suppressed through a variety of strategies [93], the latter comprising (i) structural changes in the chemistry of antibiotics to lessen their affinity to the binding sites of the transporter [94], (ii) the use of bacterial membrane permeabilizers to artificially enhance the level of antibiotics in the cell; such an innovative concept was demonstrated in *P. aeruginosa* efflux machinery [95,96], (iii) down-regulation of the expression of the EP gene resulting in a decrease in efflux complexes active in bacterial envelope [97], (iv) suppression of the power source of the drug transporter, where KCN (potassium cyanide) and CCCP (carbonyl cyanide m-chlorophenylhydrazone) interfere with the bacterial membrane to alter the energy level and lower the effective extrusion of a variety of substances, (v) shutting down the operational assembly of efflux pathways [98], (vi) Inhibitor design to covalently dock with the substrate binding sites or obstruct the canal of the antibiotic carrier pumps; several natural occurring agents as well as nanoparticles [99,100] have been shown to inhibit bacterial EPs through a “molecular plug” mechanism, (vii) providing a dummy substance to act as a competitive blocker of in-pump antibiotic transport; these various approaches to EP inhibition are summarized in Figure 4. 

An aggravating factor in the AMR challenge is the emergence and spread of resistance at a faster rate than novel drug development. Instead, the number of developed and approved antibiotics has declined by more than half over the past three decades [3], prompting a more pressing global need for innovative antimicrobial agents or successful approaches. Some such emergent strategies include bacteriophages, modified oligonucleotides with antibacterial properties, bacterial virulence blockers, and the CRISPR-Cas9 strategy [101,102]. Alongside this, additional exciting concepts involving antimicrobial peptides (AMPs), lysins, and probiotics are currently in different phases of development [102]. Altogether, though multiple options do indeed occur in nature, the greatest hurdle is to prove their effectiveness and usability in both humans and animals [103]; these novel therapeutics are not designed to kill bacteria, instead, they selectively disarm them, thereby providing a means for the antibiotic to get in and complete the job; they re-sensitize the bacteria and reinstate the efficacy of the antibiotics. Some of those strategies to stop MDR are further discussed in the next sections, the emphasis being placed on potent EP blockers.

### 3.1. Antibiotic Resistance Breakers to Stop Active Efflux in Bacteria: The Efflux Pump Inhibitors and Membrane Permeabilizers

Chemical agents that, when coupled with current antibiotics, can prevent bacterial resistance from developing are considered “antibiotic-resistance breakers” (ARBs). Any commercially available drug has the potential to do this [104]. While some ARBs have previously been used in the clinic, for example, β-lactamase inhibitors (BLIs), to date, we anticipate that the expanded area of research on ARBs can still elicit a broader range of more potent therapeutics than has been accomplished. ARBs have the potential to control deadly infectious diseases by re-establishing the efficacy of antibiotics that have been compromised; these new agents, some structurally similar to existing antibiotics, are being screened for their capacity to create “strong synergy” with established antibiotics [105]. The ARBs can either exhibit a direct bacterial inhibitory effect or not and can be used either co-administered or in combination with deficient antibiotics. The principle of bi-therapy, which has proven useful in the past by having either synergistic or additive qualities of specific antibacterial agents, is the incentive for co-administration of ARBs with standard antibiotics [106] and there has been long-term clinical use of various ARBs, most notably the BLIs [107]. A successful co-administration of ARBs is expected to augment the actions of antibiotics by counteracting the mechanisms of bacterial resistance used against them, thereby permitting the use of smaller dosages of antibiotics. A valuable approach in this regard is the MIC [108]; the most potent ARBs achieve a significant decrease in the MIC of antibiotics when compared to monotherapy; this is an intriguing perspective, both because decreasing the selection pressure of antibiotics would potentially delay the appearance of AMR, as well as expand the therapeutic window to attenuate the adverse effects experienced by patients on a single antibiotic therapy. Though ARBs have been earlier called antibiotic adjuvants, the latter refers also to additional therapies such as agents that boost host immunity to assist in the elimination of bacterial infection [109]. Therefore, this current review will be limited to an examination of compounds used to invert the mechanisms of bacterial resistance, emphasizing EP-mediated resistance. Accordingly, although enzyme-modifying inhibitors are worthy of note, we focused on two major classes of ARBs namely EPIs and membrane permeabilizers.

#### 3.1.1. Efflux Pump Inhibitors

As detailed in the previous sections, bacterial MDR is extensively promoted by EPs. Consequently, the development of innovative strategies becomes critical to overcoming AMR. Several therapeutic concepts can be applied for inhibiting or circumvent the activity of EPs, such as reducing the affinity of the antibiotic to bind to the drug transporter by chemically restructuring the drug, improving the permeability of the OM to obtain higher intracellular levels of the drug, blocking or neutralizing EP-related genes, altering ATP energy delivery, or designing competitive EPIs [110]. The latter appears to be the most successful approach to fighting bacterial efflux machinery. Most EPIs studied so far act by physically preventing the substrate molecules from passing through the transporter. However, without compounds able on their own to modify their target pumps covalently, this approach supports a certain extent of binding competition between the substrate antibiotic and the inhibitor, leading to potentiation levels [111]. In addition, antibiotics that are covalently derivatized with an EPI feature might be expected to be conventional pump blockers free from the disadvantage of competitive binding and therefore better able to improve the efficacy of the parental antibiotic [112]. Several known inhibitors have been uncovered through computer analysis or extraction from plants. In recent years, extensive reviews have been published, emphasizing the diverse array of EPI agents unveiled so far originating from natural sources (plants and microbes), chemical synthesis, and drug repurposing of previously-approved drugs [8,18,111,112,113,114]. Some of the well-established EPIs from various sources (Table 1) are described in the next sections. At the time of writing, no EPI received formal approval for use clinically.

*(i) Naturally occurring efflux pump inhibitors.* Plants have been the source of most EPIs reported. Due to their abundance of structurally diverse secondary metabolites, they are also regarded as the primary future source of new bioactive molecules against MDR. Alkaloids are among the most numerous groups of phyto-EPIs. Some of the well-known plant alkaloids with EPI function include reserpine (**1**) (an indole alkaloid from *Rauvolfia* sp.), piperine (**2**) (extracted from *Piper* sp.), and berberine (**3**) from *Berberis* sp. The effectiveness of 1 has been shown to work against NorA in *S. aureus* and Bmr EPs in *Bacillus subtilis* [115,116]. *S. aureus* strains have been re-sensitized to ciprofloxacin once combined with 2 [117]. The EPI potential of farnesol (**4**), acyclic sesquiterpene alcohol derived from a variety of dietary and aromatic plants, was assessed and found effective on *E. coli* and *S. aureus* [118]. The results of the work performed by Stapleton and co-workers [119] on green tea extracts (*Camellia sinensis*) reported the ability of two isolated compounds, epicatechin gallate (**5**) and epigallocatechin gallate (**6**), to significantly deplete oxacillin MICs against bacteria expressing MDR phenotypes. A similar effect was noted when combined with a fluoroquinolone antibiotic, norfloxacin, in *S. aureus* and *S. epidermidis* [120]. Furthermore, despite a weak inhibitory effect on NorA pumps, these catechin gallate class compounds displayed a significant ability to block Tet(K) pumps expressed in *S. aureus* and *S. epidermidis*, thus contributing to reversing tetracycline resistance. In all studies involving these two components, 5 was found to be the most active. Msr(A) and Tet(K) pumps of *S. aureus* were blocked by carnosic acid (**7**) and carnosol (**8**) (both isolated from *Rosmarinus officinalis*), two compounds of the abietane diterpene class, in combination with two antibiotics, erythromycin, and tetracycline [121]. Another potent plant-EPI is baicalein (**9**), a methoxylated flavone from *Thymus vulgaris*. Investigations on methicillin-resistant *S. aureus* (MRSA) isolates depicted significant enhancement of the MICs of tetracycline as well as β-lactam antibiotics including ampicillin and oxacillin. *Berberis* plants offer a substantial range of efficient EPIs. Among them, compound 3 has been widely studied. The inhibition of MexXY-OprM pump has been obtained with the combination of 3 and imipenem against a major pathogenic bacterium of concern, *P. aeruginosa* [122]. Porphyrin pheophorbide A (**10**) is another heterocyclic macrocycle isolated from the same plant; it can act by increasing the responsiveness of *S. aureus* to 3 and inhibiting the NorA pump [123]. Likewise, 5′-methoxyhydnocarpin (11), a flavonolignan from *Berberis* sp., has been revealed to block the NorA efflux system. Also, **11** displayed a synergistic effect with a fluoroquinolone (norfloxacin) against *S. aureus* [124]. A potential to interact synergistically has been reported with the homoisoflavonoid bonducellin (**12**), extracted from the root part of *Caesalpinia digyna*, in association with ethidium bromide to combat hard-to-treat *Mycobacterium smegmatis* [125]; these are not an exhaustive list of plant-derived EPIs, further naturally-occurring EPIs originating from plants have been extensively reviewed in some of our recent studies [8,18]. 

**Figure 5:**
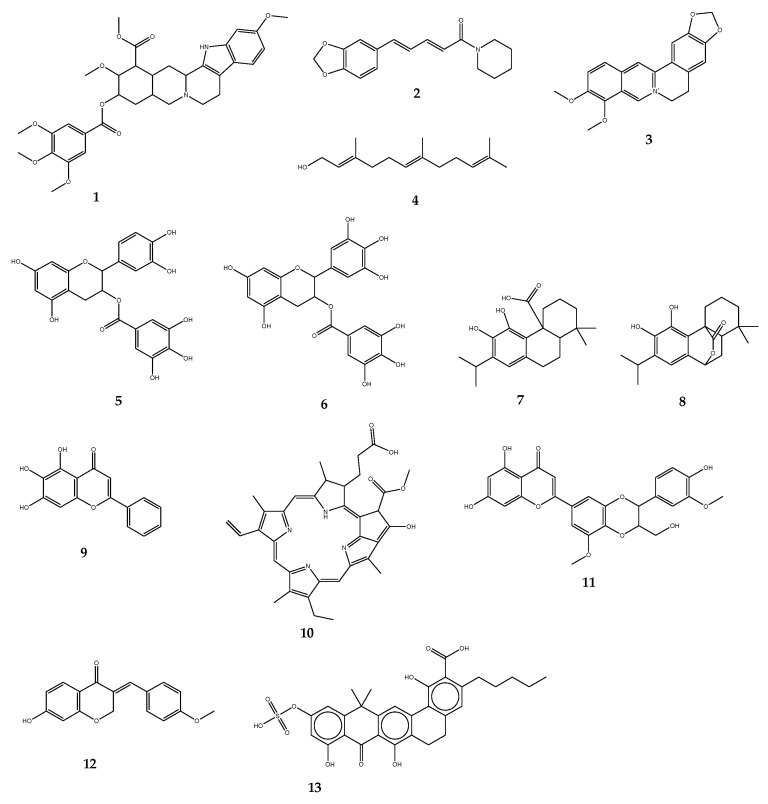
Chemical structures of some naturally-occurring efflux pump inhibitors. Reserpine (**1**), Piperine (**2**), Berberine (**3**), Farnesol (**4**), Epicatechin gallate (**5**), Epigallocatechin gallate (**6**), Carnosic acid (**7**), Carnosol (**8**), Baicalein (**9**), Porphyrin pheophorbide A (**10**), 5′-methoxyhydnocarpin (**11**), Bonducellin (**12**), EA-371α (**13**).

Besides plant-derived constituents, EPIs from natural origin have been explored using microbial products. So far, two compounds EA-371α (**13**) and EA-371δ have been extracted from *Streptomyces*. Combined with a quinolone antibiotic (levofloxacin), the two microbial compounds substantially improved the MIC of the antibiotic. The test has been performed against *P. aeruginosa* overexpressing the MexAB-OprM efflux machinery [126]. As far as it can be verified, few EPIs have been reported against Gram-negative pathogens; this is likely due to the structural complexity of their EP system and particularly the tripartite organization, involved in the efflux of a very broad range of antibacterials. NorA appears to be the most studied, especially in *S. aureus*. The structures of compounds **1**–**13** have been depicted in Figure 5. 

(*ii*) *Established efflux pump inhibitors from chemical synthesis.* In addition to naturally occurring, a range of chemically synthesized classes of EPIs have been unveiled. The peptidomimetic compounds represent one of the first groups of synthetic EPIs, the most studied member being Phenylalanine-arginine β-naphthylamide (PAβN). PAβN was found to restore the efficacy of fluoroquinolone against *P. aeruginosa*. For instance, the compound led to an outstanding decrease in the MIC of levofloxacin against *P. aeruginosa* strains. The increased effect has been noted in the strains which overexpressed the tripartite MexAB-OprM EPs [127]. The activity of PAβN and most importantly its application in the clinical setting have been hindered by their cellular toxicity. Interestingly, advanced research permitted the production of enhanced analogs (lower toxicity and serum-free drug clearance), known as MC-004124 [128]. A recent investigation predicted how PAβN acts. According to Jamshidi et al. [129], the PAβN attaches to the non-polar distal binding pocket whereby maintaining the binding monomer in the binding configuration, leading to the prevention of the pump from moving through the series of conformational changes required to meet substrate efflux. Other classes of synthetic EPIs comprised quinoline and pyridoquinoline derivatives. Both have been proven to impair the extrusion of a variety of antibiotics including norfloxacin, ciprofloxacin, and chloramphenicol in *Klebsiella aerogenes* (previously known as *Enterobacter aerogenes*) expressing MDR. Further is the 1-(1-Naphthylmethyl)-piperazine, an arylpiperazine derivative that was proven to restore the efficacy of antibacterial agents, particularly levofloxacin, in laboratory collection of *E. coli*, through inhibition of both AcrAB and AcrEF efflux systems. Similar responses have been recorded in many other Enterobacteriaceae namely *K. aerogenes*, *K. pneumoniae*, *Vibrio cholera*, and *A. baumannii* [130,131,132,133]. Unfortunately, the application of aryl piperazine derivative as EPI in humans is restricted due to their serotonin agonist features [123]. D13-9001, a pyridopyrimidine derivative was revealed to inhibit AcrB and MexB, respectively characterized in *E. coli* and *P. aeruginosa*. The product was originally designed by the Japanese company Daiichi Pharmaceutical Co., however, results from the clinical assessment are yet to be made public [114]. High-throughput screening for low molecular weight compounds unveiled MBX-2319, a potent pyranopyridine derivative, effective to block AcrAB pump [134] thereby improving the efficacy of fluoroquinolone antibiotics (ciprofloxacin, levofloxacin) and piperacillin, a penicillin beta-lactam antibiotic [93,135]. MBX-3796, a pyranopyridine of the second generation, has been developed following the SAR (structure-activity relationship) analysis. The compound may have fewer toxic effects at therapeutic doses and bypass some of the challenges with EPI, which is the PK (pharmacokinetic) profile. Indeed, intravenous administration (10 mg/kg) of MBX-3796 is well tolerated and the findings also depicted a prospective PK profile with an area under the curve (AUC) ∼10,000 and clearance (CL) below 1000 mL/hr/kg [134]. The latest advances in the pyranopyridine derivative displayed another agent, MBX-4191, with inherent antibiotic properties and noticeable synergistic potential of antimicrobials in Enterobacteriaceae. A lower effect is observed in non-fermenting GNB owed to limited permeation of OM [136]. Two compounds formerly developed to fight cancers have been reported as potent EPIs against *S. aureus*, by improving the activities of some widely used antibiotics including ciprofloxacin, tetracycline, and gentamicin; these compounds are VX 853 and VX 710, respectively called timcodar and biricodar. The further report depicted that timcodar can potentiate rifampicin, moxifloxacin, and bedaquiline, some of the drugs used to manage tuberculosis [137].

(*iii*) *Efflux pump inhibitors from previously-approved Drugs.* The concept is considered drug repurposing or drug repositioning, which is an approach that lies in the identification of alternative uses for approved drugs [138]. The use of previously-approved drugs such as ARBs is an attractive option [139]. Some examples of successful findings from drug repurposing comprised the association of trimethoprim and sertraline, which enhanced the activities of three mainstream antibiotics namely piperacillin, levofloxacin, and meropenem against *P. aeruginosa* overexpressing EPs. The potentiation effect has been also shown in vivo [140]. Paroxetine and fluoxetine (SSRIs) have been reported effective on ABC-type NorA and Bcr/CflA efflux systems of *S. aureus* and *Proteus mirabilis*, respectively [141,142]. Paroxetine displayed substantial MIC reduction in both norfloxacin and ethidium bromide [141]. Omeprazole and lansoprazole, two well-known proton pump inhibitors (PPIs) showed inhibition of the NorA efflux system in *S. aureus*; these PPIs were revealed to act synergistically with some conventional fluoroquinolones (ciprofloxacin and norfloxacin) against *S. aureus* overexpressing NorA [143]. Another common medication, verapamil, a calcium channel blocker used in the clinic to manage heart conditions has also been established as an ABC inhibitor. Several antibiotics have been potentiated once combined with verapamil against a high-risk strain of *M. tuberculosis* [144,145]. Some other widely used antipsychotic drugs, namely chlorpromazine and prochlorperazine inhibited the *S. aureus* NorA pump [146].

#### 3.1.2. Membrane Permeabilizers

Intrinsically, as opposed to GPB, GNB naturally withstands numerous antibiotic classes due to the existence of an OM, which is not permeable to these antibiotics [147]. Along with the direct damage to the cell membrane, a range of other approaches to increase the rates of antibiotic uptake into bacterial cells have been suggested [148], including among others membrane permeabilizers. As the name implies, the latter increases the permeability of the Gram-negative OM thereby facilitating the increased entry (influx) of antibiotics. Membrane permeabilizers can work through chelating and the removal of positively charged divalent ions from the OM and/or (applicable for a net cationic charge permeabilizer) by pairing with the negatively charged OM to perturb it, thus disrupting the underlying OM structure [149]. Putative membrane permeabilizers can be evaluated for their efficacy by monitoring the level of incorporation of materials that ordinarily would be unlikely to permeate the OM of GNB, for example, a hydrophobic probe; this is accomplished with NPN (*N*-phenyl-1-napthylamine), a fluorescent dye. Greater fluorescence indicates increased NPN absorption into the causative agent’s OM and, as a result, higher OM porosity [150]. Besides providing an augmented antibiotic influx, membrane permeabilization by itself may be sufficient to elicit bacterial lysis [149].

### 3.2. Nanoparticle’s Carriers

To overcome the permeability barrier frequently observed in GNB, a novel medication delivery platform based on nanocarriers can be used. Nanocarriers can also serve to specifically release high antibiotic concentrations at the local level, thereby obviating the systemic secondary effects. Strategies to deliver antibiotics such as antimicrobial polymers, nanoparticles and liposomes have been explored; its success was limited with these technologies, but continued efforts and progress are likely to make nano-delivery an integral component in defeating antibiotic resistance [151].

### 3.3. Biologics

Biologics (biological drugs) are compounds made from living organisms or contain components of living organisms; these medications target specific genotypes or protein receptors [152]. Beyond small compounds, biopharmaceuticals and associated technologies are critical to the development of future ARBs. Scientists should explore the potential benefits of using biological drugs in targeted therapy to overcome resistance and reduce the selection pressure associated with wide-spectrum, off-target antibiotics. The success of antibody-drug combinations in the therapy of cancer has spurred interest in antibody-antibiotic conjugates involving specific antibodies to bacteria [153], likewise, Lehar and co-workers provided evidence of preclinical success in addressing intracellular *S. aureus* [154].

### 3.4. Bacteriophage Therapy

Also noteworthy is phage therapy (PT), an approach involving the use of viruses that selectively infect bacterial cells; although its creation and deployment precede those of modern antibiotics, ambiguity about the efficacy of phage-based formulations led to their replacement by the latter [155]. Currently, the application of therapy involving phages is not widespread and is only authorized in a limited number of countries [155], however, there is evidence of its usefulness in the management of *E. coli* infections [156], *P. aeruginosa*, *A. baumannii* [157], and *K. pneumoniae* [158] in mice, and there have been several phage formulations in Phase I/II clinical trials [109].

### 3.5. Bacteriocins

The review by Telhig and co-workers provided an appraisal of bacteriocins, emphasizing their application as tools to counter bacterial resistance, particularly in hard-to-treat Gram-negative microorganisms [103]. Bacteriocins are a broad category of peptides with antimicrobial properties (AMPs) that originated from bacteria; they are classified into two major categories, unmodified and/or modified peptides. The modified peptides are members of a broad family of ribosomally generated and post-translationally modified peptides (RiPPs), that were obtained primarily from microorganisms as well as animals and plants [159,160]. Recognized as in vitro pathogen inhibitors, several bacteriocins exhibit high levels of specific activity against clinical strains, including those resistant to antibiotics [161]. Their effectiveness as pathogen and spoilage inhibitors has been extensively researched. As a result, it is largely acknowledged that some could be utilized for medicinal purposes and as a viable substitute for mainstream antibiotics [162,163]. When produced by enterobacteria, bacteriocins are termed as microcins. The latter are small (<10 kDa) modified or unmodified peptides [164]. Microcins serve important functions in the bacterial environment, specifically a key function in microbial competition [165,166]. The MIC of bacteriocins generally ranged from nanomolar to micromolar, suggesting potent activity. Furthermore, they generally and specifically target GNB, indicating their narrow spectra of activity [164,165]. To achieve their competitive roles, bacteriocins have a similar pathway to enter their bacterial targets, hacking into the nutrient uptake pathways of phylogenetically related bacteria that are competing for the same resources. The pathway for iron import is the most frequently assaulted [164]. Once within bacteria, microcins will interfere with and perturb several diverse bacterial functions, including transcription [167], translation [168], DNA structure [169], mannose transport [170], energy production or cell envelope function [171]. Owing to their specific features and complex modes of action, microcins are thought to be a feasible option as an antibiotic substitute, aiding in solving the pressing challenge of AMR [161,172,173]. Because of their tight spectrum of inhibition, the inherent value of these would be fewer adverse effects than antibiotics, maintaining the diversity of the microbiota and downplaying the chance of spreading resistance [103].

### 3.6. De Novo Strategies

Taking on a part for *de novo* techniques in the advancement of ARBs is expected. The computational modeling of specific biological targets and systems is a field that should drive the further advancement of future ARBs by expanding the fundamental understanding of the operating mechanisms of ARBs. Notably, this is especially relevant for efflux inhibition, wherein, without crystal structures (given the complex and transmembrane nature of bacterial efflux proteins), it is crucial to use computer processing capacity to establish a mechanistic understanding of efflux inhibition [129,174]. The state-of-the-art currently requires a trade-off between both precision and computational load. Protein-scale systems are being modeled using coarse-grained molecular DS (dynamic simulations), using accurate and resource-intensive quantum mechanical optimizations restricted to only small areas of them [175]. Yet, with mounting excitement and interest in the much-touted quantum computing technology [176], the processing power improvements necessary for quantum mechanical simulations to be executed on protein-sized devices could be realized. Such breakthroughs could enable a far more refined package of in silico modeling technologies to ensure the future development of antibiotics and ARB candidates for clinical trials.

## 4. Obstacles in the Development of AMR Blockers: The Case of Efflux Pump Inhibitors

To qualify as clinically competent, EPIs must demonstrate effective therapy at an achievable serum/tissue level with the lowest toxicity. Because they are considered combination therapy, EPIs should indeed work in concert with their co-administered antibacterial to be more effective than the medicines taken alone [16]. Notwithstanding the immense potential of the numerous surveys relating to EPIs, the design of an approved and effective EPI has proven to be a very difficult task. The reasons are ascribed to some factors including the structural heterogeneity of EPIs, the broad substrate specificity of MDR pumps, and off-target toxicity [177]. A significant fraction of experimental EPIs is most usually discovered by chance during the screening of huge pools of candidates having antagonistic effects towards EPs in vitro. High-throughput screening (HTS) and SAR analyses have remained the most effective methods for identifying new EP blockers [16]. Some promising techniques, including electron cryo-microscopy (cryoEM) and AphaFold have had a major impact on the study of membrane proteins that form the basis of many resistance mechanisms, including EPs. Yet, such pumps are intrinsically multiple-component membrane proteins, and the crystallization of intricate macro-molecular structures has frequently proved to be exceedingly difficult [178]; this hindered both our understanding of EP functionality and defining their underlying substrate patterns, subsequently impeding the design of target-specific and efficient EPIs. 

A feasible approach to developing EPIs may involve testing previously-approved drugs; in this way bypassing the challenges involved in the process of identifying and developing novel drug candidates. Nonetheless, many medications are likely to be hazardous at doses sufficient to impede efflux, as evidenced by PAβN, reserpine, verapamil, and more [179]. However, because of non-targeted effects, inadequate strength, a weak PK/PD pattern, and a record of cell toxicity in humans, the practical application of these potential EPIs has remained elusive [16]. EPIs, which could be used clinically as an adjuvant to antimicrobial treatment have had very limited effectiveness. Despite some hopeful findings for EPIs of natural origin [113]. Given the scarcity of reliable experimental models, there has been minimal effort to enhance the vast number of chemical compounds discovered in plant sources through medicinal-chemistry optimization; this difficulty in developing EPIs and similar computer simulations might be attributed to a research gap in the operational assemblages of various EPs, as well as an absence of trustworthy biochemical, computational, and structural models of EP activities [180].

As such, what’s the deal with the lack of creativity? Developing a novel formulation is a cost-effective investment. That’s why the concept of repurposing medications that have previously been licensed for therapeutic use is appealing. Inventing new antibiotics is much more costly than re-sensitizing the microorganisms. Developing EPIs that effectively block RND efflux systems has proved challenging owing to the complex structure and poly-selective characteristics of such pumps, yet there is still a need to understand the working mechanisms of existing EPIs and to improve our comprehension of the complex structure of efflux systems of RND family to ensure that they are effective and efficient in their application. 

Figuring out the inhibition mechanisms of EPs by performing long-term molecular dynamics (MD) as well as quantum molecular dynamics (QMD) simulations may make significant progress in the area of the discovery of EPIs. Long-term MD simulations of pre-developed EPIs like PAβN and other bacterial EPs that are possibly inhibited by these EPIs are now being used to identify undiscovered binding sites. Furthermore, these simulations can provide useful insights into the detection of diverse transporter-binding protomers. More work is needed to co-crystallize EPIs and EPs and uses these crystal structures to further build robust and operational molecular models that can take use of the most recent breakthroughs in high-performance computing. Using the concepts of a rational medication design approach and examining both naturally-occurring and synthetically manufactured candidates, more clinically relevant EPIs may be developed and might be a key weapon in the fight against AMR [114].

The major challenge with reserpine, consistent with that of many other EPIs, is toxicity to cells in mammals. It was proven that reserpine induces central nervous system disorders [181], restricting it from being used as an ARB in practice. Future investigations in the area should target derivatives with enhanced toxicological patterns as many of the compounds reported are not suitable for further clinical development based on this fact [128,149]. To this extent, the study of polymyxin B derivatives with lower kidney toxicity [182,183] and the constant improvement of the current EPI scaffolds appear to be promising. [93,134,135].

## 5. Concluding Remarks

Antimicrobial resistance (AMR) is spreading at an unprecedented level, and it is currently universally acknowledged as a worldwide problem that demands immediate response. Efflux pump-mediated resistance is the leading strategy developed by bacteria to overtake the antibiotic effects. A significant range of approaches is available to stop efflux resistance, with varying degrees of effectiveness. The antibiotic resistance breakers (ARBs) provide a viable area of study to address this. Among ARBs, efflux pump inhibitors (EPIs) and membrane permeabilizers appear to be the principal weapons for impairing the efflux system machinery. Though the application of EPIs as medications is fraught with difficulties, this shouldn’t in any way negates the significance and benefits they provide. The traditional ARB strategy of combining separate antibiotics with ARB agents to increase the original effect should be reconsidered; it undeniably provides benefits, the most notable of which are the underlying flexibility of both the combination therapies and the prospect of synergistic interaction between the two drugs. However, these features are outweighed by a variety of issues, albeit with an additional compliance cost as a consequence of combining medications and a need that somehow the pharmacokinetic properties of the treatments be equivalent. Besides EPIs, promising approaches are explored, including the development of nanoparticle carriers, biologics, bacteriocins, and computational modeling of specific biological targets and systems. These approaches offer the opportunity to produce innovative agents that can selectively and efficiently block the efflux pump machinery.

## Figures and Tables

**Table 1:** Target pumps, and characteristic antibiotic substrates of some established efflux pump inhibitors from various sources.

Efflux Pump Inhibitors	Origin	Target Pumps (Bacteria)	Antibiotic Substrates	References
Reserpine	*Rauvolfia* sp.	NorA, Bmr, TetK, LmrA, PmrA, MepA (*B. subtilis, S. aureus, S. pneumoniae, Lactococcus lactis)*	Norfloxacin, ciprofloxacin, tetracycline	[115,116]
Piperine	*Piper* sp.	NorA, MdeA, Rv1258c (*E. coli*, *S. aureus*, *Mycobacterium* *spp.*)	Ciprofloxacin, norfloxacin	[117]
Berberine	*Berberis* sp.	MexAB-OprM, NorA (*P. aeruginosa*, *S. aureus*)	Imipenem	[122]
Epicatechin gallate	*Camellia sinensis*	NorA, TetK (*S. aureus*, *S. epidermidis*)	Oxacillin, norfloxacin	[119,120]
Epigallocatechin gallate	*Camellia sinensis*	NorA, TetK (*S. aureus*, *S. epidermidis*)	Oxacillin, norfloxacin	[119,120]
Carnosic acid	*Rosmarinus officinalis*	NorA, MsrA (*S. aureus*)	Erythromycin, tetracycline	[121]
Carnosol	*Rosmarinus officinalis*	NorA, MsrA (*S. aureus*)	Erythromycin, tetracycline	[121]
Baicalein	*Thymus vulgaris*	NorA, TetK (*S. aureus*, *Salmonella enteridis*, *E. coli*)	Tetracycline, ampicillin, oxacillin, ciprofloxacin	[18]
Porphyrin pheophorbide A	*Berberis* sp.	NorA (*S. aureus*)	Berberine	[123]
5′-methoxyhydnocarpin	*Berberis* sp.	NorA (*S. aureus*)	Norfloxacin	[124]
EA-371α	*Streptomyces*	MexAB-OprM (*P. aeruginosa*)	Levofloxacin	[126]
EA-371δ	*Streptomyces*	MexAB-OprM (*P. aeruginosa*)	Levofloxacin	[126]
PAβN	Synthetic	MexAB-OprM (*P. aeruginosa*)	Levofloxacin	[127]
1-(1-Naphthylmethyl)-piperazine	Synthetic	AcrAB, AcrEF (*E. coli*)	Levofloxacin	[130,131,132,133]
D13-9001	Synthetic	AcrAB-TolC (*E. coli*), MexAB-OprM (*P. aeruginosa)*	Wide variety	[114]
MBX-2319	Synthetic	AcrAB (*E. coli*)	ciprofloxacin, levofloxacin, piperacillin	[93,134,135]
Trimethoprim, sertraline	Drug repurposing	Efflux systems of *P. aeruginosa*	piperacillin, levofloxacin, meropenem	[140]
Paroxetine, fluoxetine	Drug repurposing	NorA, Bcr/CflA (*S. aureus*, *Proteus mirabilis*)	Norfloxacin, ethidium bromide	[141,142]
Omeprazole, lansoprazole	Drug repurposing	NorA (*S. aureus*)	Ciprofloxacin, norfloxacin	[142]
Verapamil	Drug repurposing	ABC (*M. tuberculosis*)	Bedaquiline	[144,145]
Chlorpromazine, prochlorperazine	Drug repurposing	NorA (*S. aureus*)	-	[146]
Biricodar, timcodar	Synthetic/Drug repurposing	EtBr efflux (*S. aureus*, *E. faecalis*, *S. pneumoniae*)	Ciprofloxacin, tetracycline, gentamicin, rifampicin, moxifloxacin, bedaquiline	[137]

Note: ABC, ATP Binding Cassette; EtBr, Ethidium Bromide; PaβN, Phenylalanine-arginine β-naphthylamide.

## Data Availability

Not applicable.

## Abbreviations

AA	Amino Acid
ABC	ATP Binding Cassette
AMP	Antimicrobial Peptide
AMR	Antimicrobial Resistance
ARB	Antibiotic Resistance Breaker
CCCP	Carbonyl Cyanide m-Chlorophenylhydrazine
DS	Dynamic Simulation
EP	Efflux Pump
EPI	Efflux Pump Inhibitor
GNB	Gram-Negative Bacteria
GPB	Gram-Positive Bacteria
HTS	High-Throughput Screening
MATE	Multidrug and Toxin Extrusion
MD	Molecular Dynamics
MDR	Multidrug Resistance
MFP	Membrane Fusion Protein
MFS	Major Facilitator Superfamily
MIC	Minimal Inhibitory Concentration
OM	Outer Membrane
OMP	Outer Membrane channel Protein
PACE	Proteobacterial Antimicrobial Compound Efflux
PCR	Polymerase Chain Reaction
PPI	Proton Pump Inhibitor
PT	Phage Therapy
QMD	Quantum Molecular Dynamics
RiPP	Ribosomally generated and Post-translationally modified Peptides
RND	Resistance Nodulation Cell Division
ROS	Reactive Oxygen Species
SAR	Structure-Activity Relationship
SMR	Small Multidrug Resistance
WHO	World Health Organization
